# Potential improvement in spatial accessibility of methadone treatment with integration into other outpatient substance use disorder treatment programs, New York City, 2024

**DOI:** 10.1371/journal.pone.0317967

**Published:** 2025-02-05

**Authors:** Marcus A. Bachhuber, Chinazo O. Cunningham, Ashly E. Jordan

**Affiliations:** 1 New York State Office of Addiction Services and Supports, New York, New York, United States of America; 2 Department of Medicine, Oregon Health & Science University, Portland, Oregon, United States of America; University of New Mexico Health Sciences Center, UNITED STATES OF AMERICA

## Abstract

**Background:**

Methadone is an effective treatment for opioid use disorder; however, its provision in the US is limited to federally-regulated opioid treatment programs (OTP). Expansion of methadone treatment into non-OTP substance use disorder (SUD) treatment programs (‘expanded methadone treatment access’) is a promising intervention to increase access.

**Methods:**

We performed a cross-sectional geospatial analysis of public transit times to OTPs, expanded methadone treatment access, and other healthcare facilities as of March, 2024 in New York City (NYC). We estimated one-way public transit travel time and compared travel times using population weighted paired t-tests.

**Results:**

For OTPs, 38.2% (95% CI: 38.0, 38.4) of the NYC population was within 15 minutes and 79.7% (95% CI: 79.5, 79.9) was within 30 minutes. For expanded methadone treatment access, 72.1% (95% CI: 71.9, 72.2) of the NYC population was within 15 minutes and 97.5% (95% CI: 97.5, 97.6) was within 30 minutes. The mean travel time was 20.4 minutes (SD: 10.9) for OTPs and 12.1 minutes (SD: 7.1) for expanded methadone treatment access (difference: -8.3 minutes [95% CI: -8.5, -8.1]; P < 0.001). The mean travel time for expanded methadone treatment access was slightly longer than the mean travel time for dialysis facilities (difference: 0.22 minutes [95% CI: 0.06, 0.39]; P = 0.009]), not significantly different than Federally Qualified Health Centers (difference: -0.06 minutes [95% CI: -0.22, 0.11]; P = 0.51), and significantly shorter than the mean travel time to ambulatory surgical centers (difference: -6.3 [95% CI: -6.5, -6.0]; P < 0.001) and hospitals (difference: -8.1 [95% CI: -8.3, -7.9]; P < 0.001).

**Conclusion:**

Efforts to increase access to methadone treatment in the US should promote expansion to additional non-OTP outpatient SUD treatment programs. Such integration is anticipated to increase spatial accessibility of methadone treatment substantially, greatly enhancing the potential for patient access.

## Introduction

Opioid-involved drug overdose deaths continue to be at record highs in the United States (US) and in New York State (NYS) [1]. Methadone is an effective and evidence-based medication for opioid use disorder (MOUD) that reduces illegal opioid use and is associated with substantial reductions in mortality, including an up to 50% reduction in overdose deaths [2–5]. While methadone for other indications (e.g., chronic pain) can be obtained in community pharmacies, methadone treatment for opioid use disorder is currently only available through federally-regulated specialty opioid treatment programs (OTPs) [2].

Spatial accessibility of care, the distance and travel time to a health care facility, is an important dimension of access to care [6, 7]. Across populations, including variations in substances used, spatial accessibility of SUD treatment programs is an important factor contributing to improved treatment outcomes [8–23]. For OTPs, the effect of spatial accessibility on care is magnified due to the requirement for frequent visits to receive methadone [2]. Recent regulatory changes permit more flexible methadone dosing and lower threshold service structures, allowing for more at-home methadone doses [24]. However, these flexibilities have not been extended to all patients, and most patients typically must visit OTPs at least several times per week [25]. For example, in a recent NYS analysis, over half of patients had a dosing schedule requiring multiple in-person visits per week [26].

In the US, options for expansion of methadone treatment outside of brick-and-mortar OTPs are currently limited. Under federal regulations, certified OTPs can establish a medication unit (brick-and-mortar or mobile facility) that is a part of, but geographically separate from, the OTP [24]. Despite recent regulatory changes and clarifications to this process, significant administrative and logistical barriers to widespread adoption persist [27]. Outside of OTPs, in previous US studies, expansion of methadone treatment to pharmacies and Federally Qualified Health Centers (FQHC) was estimated to reduce travel times [28–31]; however, such an expansion of methadone treatment outside of the context of an OTP would require additional major federal regulatory changes, potentially from legislative action, as a prerequisite for implementation. The end result of these limited options for expansion of methadone treatment is a pronounced shortage in the US [32].

Until methadone treatment can be provided at facilities other than OTPs, integrating methadone treatment into non-OTP outpatient SUD treatment programs is a promising policy intervention to expand access. Supporting non-OTP outpatient SUD treatment programs to become certified as an OTP and provide methadone treatment leverages pre-existing resources and expertise. In NYS, the Office of Addiction Services and Supports (NYS OASAS) recently made available more than $26 million in funding to help integrate methadone treatment and other outpatient SUD treatment services within one setting [33–35]. Such funding can help overcome initial barriers to providing methadone treatment, such as purchasing dispensing equipment.

The aims of this study were twofold: 1) to estimate the spatial accessibility of OTPs alone and in comparison with the spatial accessibility of other health care facilities in New York City (NYC) and 2) to examine potential changes in spatial accessibility of methadone treatment with integration of methadone treatment into additional non-OTP outpatient SUD treatment programs. While previous studies of spatial accessibility of SUD treatment have often examined drive times [14, 23, 28–31, 36–39], such methods may not realistically estimate travel times in highly urbanized areas where residents rely on public transit [40]. For example, in NYC, approximately 56% of residents rely on public transit to commute to work [41]. Therefore, the current study examines spatial accessibility through estimation of travel times via public transit.

## Methods

We conducted a cross-sectional analysis of public transit accessibility of OTPs and made comparisons with other health care facilities in NYC. To estimate the impact of expanded methadone treatment access through integration of methadone treatment into additional non-OTP outpatient SUD treatment settings, we created a group of programs that included all outpatient SUD programs as well as OTPs (‘expanded methadone treatment access’). For comparison, we also estimated public transit accessibility of other health care facilities, including dialysis facilities, ambulatory surgical centers, FQHCs, and hospitals. While these facilities clearly provide different services and have different entry and retention requirements, we selected these comparison facilities based on previous studies and potential similarities in usage patterns, as well as the need for specialized licensure, equipment, or both [28, 29]. The comparison between the accessibility of OTPs and dialysis facilities is particularly relevant, as patients with end stage renal disease and those receiving methadone treatment are frequently required to travel to the treatment site multiple times per week.

We obtained address data for all outpatient SUD treatment programs (OTPs and non-OTP outpatient SUD treatment programs) from the NYS OASAS provider and program search tool [42]. We defined expanded methadone treatment access as the sum of OTPs and non-OTP outpatient SUD treatment programs. For dialysis facilities, we obtained address data from the Centers for Medicare & Medicaid Services (CMS) provider data [43]. For ambulatory surgical centers, FQHC, and acute care hospitals, we obtained data on CMS approved facilities provided by the Health Resources and Services Administration (HRSA) [44]. For outpatient SUD treatment programs and dialysis facilities, we geocoded locations using a previously described three-step procedure in ArcGIS Pro version 3.2.2 [30]. For ambulatory surgical centers, FQHC, and hospitals, HRSA data already contained facility geocoordinates. We geocoded all facilities in NYS, which allowed for a nearest facility to be outside of NYC, if applicable. Because the focus of this analysis was on resources available within NYS, we did not include facilities in neighboring states. We obtained all facility location data as of March 2024.

We created a transit network for NYC that modeled walking and using public transit lines (bus, subway, rail). We obtained publicly available transit schedules (General Transit Feed Specification files) from the Metropolitan Transportation Authority [45] and streets data from the Census Bureau [46]. We conducted analyses at the block group level. We obtained the most recent (year 2020) geocoordinates of the centers of population of block groups from the Census Bureau [47]. Using the ArcGIS (Esri, 2023) nearest facility locator, we estimated one-way public transit travel time for all block groups using published transit timetables, inclusive of time walking and time on public transit modes of travel for all facility types. We estimated the public transit travel time for a weekday 8 am departure [48].

### Statistical analysis

First, we conducted descriptive analyses of estimated travel time by block group. We analyzed travel time as both a continuous variable and by creating different time thresholds (≤ 15, ≤ 30, ≤ 45, and ≤ 60 minutes). To create estimates that were representative for the overall NYC population, we weighted all analyses by the 2022 5-year American Community Survey block group population estimates and removed all block groups with zero population [49]. For the percentages of the population within each travel time threshold, we constructed 95% confidence intervals (CI) using the successive differences replicate method as described by the Census Bureau [50]. Next, using weighted paired t-tests, we compared travel times for OTPs and expanded methadone treatment access with travel times to other health care facilities. Based on reviewer feedback, we subsequently estimated population-weighted travel times for OTPs and expanded methadone treatment access at the 75^th^, 90^th^, 95^th^, and 99^th^ percentiles and graphed the distribution of both with kernel density estimation. A two-tailed P-value of ≤ 0.05 was considered statistically significant. As this study did not involve human subjects, it was determined to be exempt by the University of Buffalo Institutional Review Board. We prepared this manuscript in accordance with STROBE guidelines [51].

## Results

**Fig 1:**
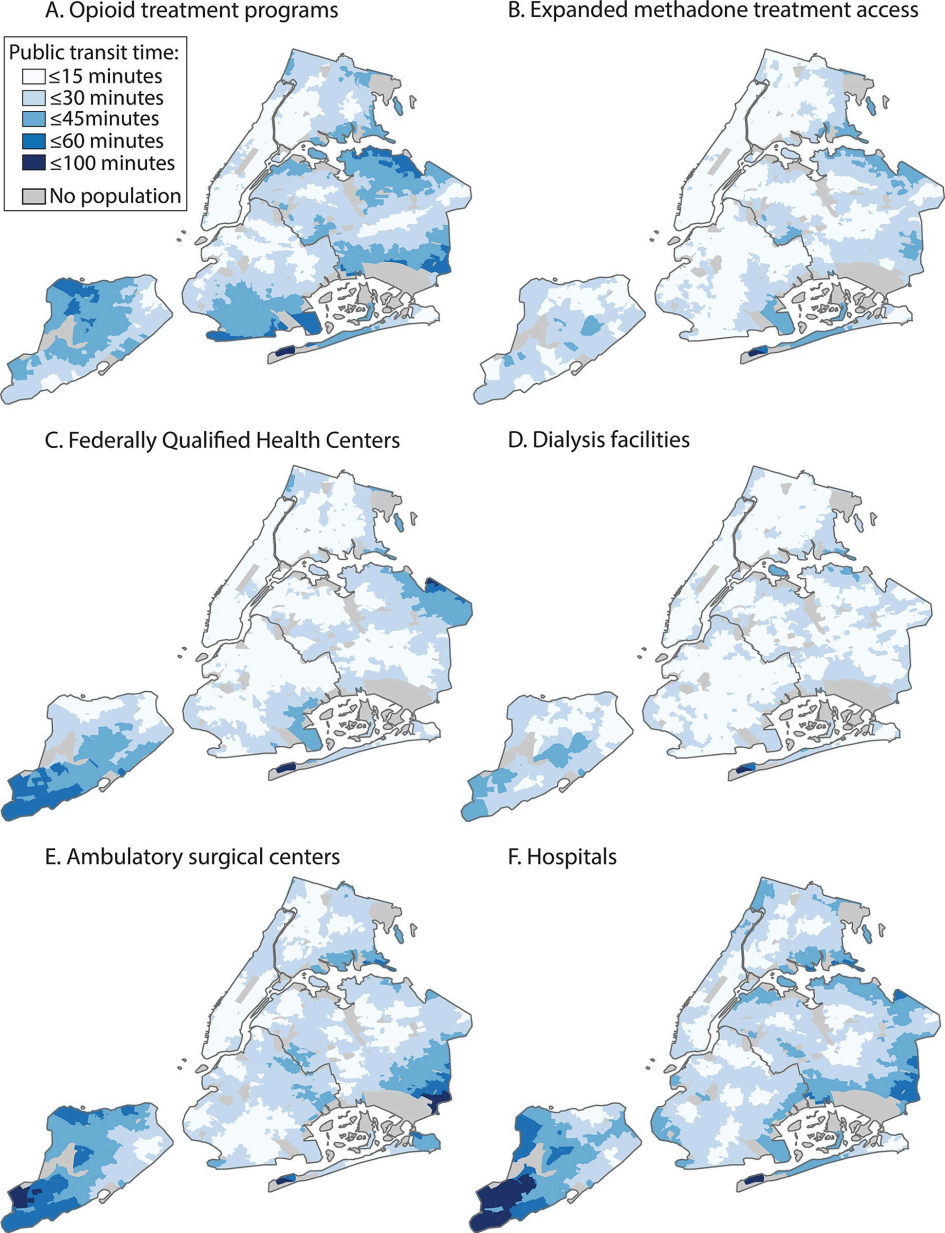
Areas within one-way public transit travel time thresholds to opioid treatment programs, expanded methadone treatment access, and other health care facilities, New York City, 2024. Note: Expanded methadone treatment access refers to all outpatient substance use disorder treatment programs including opioid treatment programs.

In NYC, we identified 6,397 block groups totaling an estimated population of 8.6 million individuals. Within the boundaries of NYC, we identified 55 OTPs, 172 non-OTP outpatient SUD treatment programs, 156 dialysis facilities, 280 FQHCs, 61 ambulatory surgical centers, and 36 hospitals. The mean travel time was 20.4 minutes (SD: 387.8) for OTPs and the mean travel time was 12.1 minutes (SD: 260.7) for expanded methadone treatment access (Table 1, Fig 1). For OTPs, 38.2% (95% CI: 38.0, 38.4) of the population was within a travel time of 15 minutes and 79.7% (95% CI: 79.5, 79.9) was within a travel time of 30 minutes. For expanded methadone treatment access, 72.1% (95% CI: 71.9, 72.2) of the population was within a travel time of 15 minutes and 97.5% (95% CI: 97.5, 97.6) was within a travel time of 30 minutes.

**Table 1:** One-way public transit travel time to opioid treatment programs, expanded methadone treatment access, and other health care facilities, New York City, 2024.

	Minutes, mean (SD)	Minutes, median (IQR)	≤ 15 minutes, % (95%CI)^a^	≤ 30 minutes, % (95%CI)	≤ 45 minutes, % (95%CI)	≤ 60 minutes, % (95%CI)
OTPs	20.4 (10.9)	18.7 (11.9, 27.6)	38.2 (38.0, 38.4)	79.7 (79.5, 79.9)	98.0 (97.9, 98.0)	100.0 (99.9, 100.0)
Expanded methadone treatment access^b^	12.1 (7.1)	10.6 (6.8, 16.0)	72.1 (71.9, 72.2)	97.5 (97.5, 97.6)	100.0 (99.9, 100.0)	100.0 (100.0, 100.0)
FQHCs	12.2 (8.7)	10.2 (6.0, 15.6)	72.4 (72.2, 72.7)	95.1 (95.0, 95.1)	99.2 (99.2, 99.3)	99.9 (99.9, 100.0)
Dialysis facilities	11.9 (5.8)	11.3 (7.7, 15.1)	74.8 (74.5, 75.0)	99.0 (98.9, 99.0)	100.0 (99.9, 100.0)	100 (100.0, 100.0)
Ambulatory surgical centers	18.4 (10.1)	16.8 (11.3, 22.8)	40.1 (39.9, 40.3)	88.2 (88.0, 88.3)	97.6 (97.5, 97.6)	99.7 (99.7, 99.8)
Hospitals	20.2 (9.9)	19.0 (13.5, 25.4)	32.1 (31.8, 32.3)	85.9 (85.7, 86.1)	97.8 (97.7, 97.9)	99.3 (99.2, 99.3)

Abbreviations: 95% CI, 95% confidence interval; FQHCs, federally qualified health centers; OTPs, opioid treatment programs; SUD, substance use disorder

^a^ 95% confidence intervals can include the point estimate due to rounding

^b^ Refers to all outpatient SUD treatment programs including opioid treatment programs

Compared with dialysis facilities (difference: 8.5 minutes [95% CI: 8.2, 8.7], P < 0.001) and FQHCs (difference: 8.2 minutes [95% CI: 8.0, 8.4]; P < 0.001), the mean travel time to OTPs was significantly longer (Table 2). Compared with ambulatory surgical centers, the mean travel time to OTPs was modestly longer (difference: 2.0 minutes [95% CI: 1.7, 2.3; <0.001]). Compared with hospitals, the mean travel time to OTPs was not significantly different (difference: 0.13 minutes [95% CI: -0.11, 0.37]; P = 0.30).

**Table 2:** Mean difference in one-way public transit travel time between opioid treatment programs, expanded methadone treatment access, and other health care facilities, New York City, 2024.

	Treatment model			
	OTPs only		Expanded methadone treatment access^a^	
Comparison facility	Difference, minutes^b^ (95% CI)	P	Difference, minutes^b^ (95% CI)	P
Opioid treatment programs	Ref.	−	-8.3 (-8.5, -8.1)	<0.001
Expanded methadone treatment access^a^	8.3 (8.1, 8.5)	<0.001	Ref.	−
Federally Qualified Health Centers	8.2 (8.0, 8.4)	<0.001	-0.06 (-0.22, 0.11)	0.51
Dialysis facilities	8.5 (8.2, 8.7)	<0.001	0.22 (0.06, 0.39)	0.009
Ambulatory surgical centers	2.0 (1.7, 2.3)	<0.001	-6.3 (-6.5, -6.0)	<0.001
Hospitals	0.13 (-0.11, 0.37)	0.30	-8.1 (-8.3, -7.9)	<0.001

^a^ Refers to all outpatient SUD treatment programs including OTPs

^b^ A positive number indicates that the one-way public transit time for the treatment model (i.e., OTPs only or expanded methadone treatment access) is longer than the one-way public transit time for the comparison facility. A negative number indicates that the public transit time for the treatment model is lower, on average, than the public transit time for the comparison facility.

95% CI = 95% confidence interval; OTP = opioid treatment program; SUD = substance use disorder

**Fig 2:**
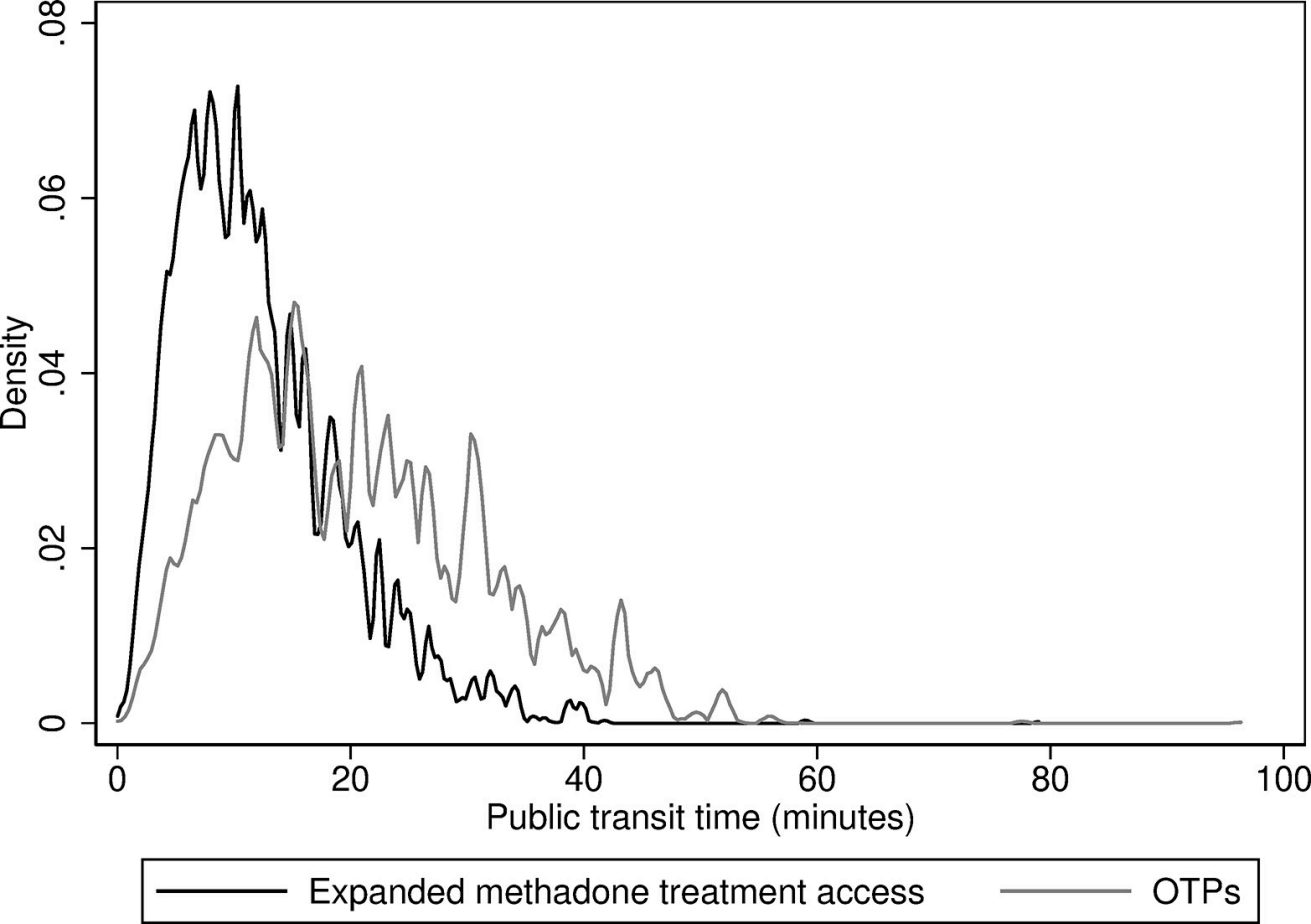
Distribution of one-way public transit travel times to opioid treatment programs and expanded methadone treatment access, New York City, 2024. Note: Expanded methadone treatment access refers to all outpatient substance use disorder treatment programs including opioid treatment programs. OTP = opioid treatment program.

Compared with OTPs, the mean travel time for expanded methadone treatment access was significantly shorter (difference: -8.3 minutes [95% CI: -8.5, -8.1; P < 0.001]). Compared with dialysis facilities (difference: 0.22 minutes [95% CI: 0.06, 0.39]; P = 0.009), the difference in mean travel time for expanded methadone treatment access was minor, with no significant difference for FQHCs (difference: -0.06 minutes [-0.22, 0.11]; P = 0.51). Comparing OTPs and expanded methadone treatment access by percentile, the travel time was lower for expanded methadone treatment access at each percentile (Table 3, Fig 2).

**Table 3:** One-way public transit travel time to opioid treatment programs and expanded methadone treatment access by percentile, New York City, 2024.

	75^th^ percentile, minutes	90^th^ percentile, minutes	95^th^ percentile, minutes	99^th^ percentile, minutes
Opioid treatment programs	27.6	35.7	40.9	47.0
Expanded methadone treatment access^a^	16.0	22.0	25.6	33.6
Percentage difference	-42.0%	-38.4%	-37.4%	-28.5%

^a^ Refers to all outpatient substance use disorder treatment programs including opioid treatment programs

## Discussion

In this cross-sectional analysis in NYC, we found that travel time to OTPs is longer than to other clinical settings that people attend with comparable frequency, such as dialysis facilities. Although 79.7% of the NYC population was within 30 minutes of an OTP, the frequent visits required for methadone treatment can create a substantial travel burden. Our findings suggest that expanding methadone treatment to additional non-OTP outpatient SUD treatment programs would substantially reduce travel time overall and for those with pre-existing long travel times to OTPs. By reducing travel time, expanded methadone treatment access would substantially increase spatial accessibility of methadone treatment and potentially improve methadone treatment outcomes.

To further understand the spatial accessibility of OTPs in NYS, we can compare our findings to travel time standards for other US agencies such as Medicaid and the US Veterans Health Administration (VA), as well as previously published research. In a recent study of 35 state Medicaid programs, 13 states had network adequacy travel time standards for managed care plans specific to SUD ranging from 20 to 110 minutes (median: 30 minutes) [52]. However, few states’ network adequacy standards accounted for use of public transit [53]. In the VA system, individuals without access to mental health care services within a 30 minute travel time or specialty services within a 60 minute travel time are potentially eligible for care at a more conveniently located non-VA community provider [54]. For the overall NYC population, access to OTPs is largely within time standards for these other agencies. However, in a recent study, an estimated drive time longer than a much lower threshold (10 minutes) was associated with significantly worse methadone treatment outcomes, suggesting that improving spatial accessibility further may improve treatment outcomes [14].

Our study adds to the existing evidence base on the potential for leveraging existing health care facilities (e.g., pharmacies and FQHC) as methadone treatment sites in the US [28–31]. While historic changes in the federal guidelines on methadone dosing at OTPs have the potential to transform methadone treatment and treatment outcomes nationally and in NYS, providing methadone treatment at other health care facilities would require major coordinated regulation changes at various federal agencies. Given this major barrier, NYS OASAS is currently supporting expansion of methadone treatment to non-OTP outpatient SUD treatment programs to increase methadone treatment access throughout the state. Until broader transformation occurs, federal, state, and local policymakers can support SUD treatment programs in integrating methadone treatment. These efforts can include providing funding for upfront costs (i.e., safes and methadone dispensing equipment), as well as technical assistance on navigating regulatory requirements, and becoming certified by the appropriate state and federal agencies.

While full integration of methadone treatment into non-OTP SUD treatment programs would have the greatest effect on spatial accessibility, other models of integration could help bridge the gap. These could include, for example, a hub-and-spoke model where methadone treatment is initiated at an OTP and then transferred to a SUD treatment program after the initial stabilization period [55]. Or, a model where the SUD treatment program serves primarily as a dispensing site only, with prescribing and management by an OTP provider. Broadly, our findings suggest that any efforts to bring methadone treatment into the broader landscape of SUD treatment programs would be anticipated to increase spatial accessibility.

Our study has several limitations. First, our findings are limited to NYC and may not be generalizable to other areas, including to other urbanized geographies. However, with a population of 8.6 million individuals, NYC is larger than all but 12 states [49]. Second, public transit time to the nearest facility may not accurately represent an individual’s experience if they have needs that cannot be met by the nearest facility, or the nearest facility does not have availability. Therefore, our estimated transit times may be underestimates for some individuals. Third, as our focus was on specialty SUD treatment programs, we did not include office-based buprenorphine prescribers. Fourth, our estimates of public transit time do not account for differences in personal preferences in mode of transportation, which can influence perceived travel time [48]. Further, we calculated travel times using transit timetables, which do not account for real world delays. Fifth, our analysis did not include accessibility features in public transit or streets and so our estimated transit times may not be representative of transit time for individuals with disabilities. Sixth, we did not have sufficient data on facility capacity to construct more complex measures of accessibility such as the enhanced two‐step floating catchment area [56]. Seventh, we used data on CMS approved or certified health care facilities which may have missed some facilities; however, we expect the number of non-CMS approved or certified health care facilities to be low as Medicare is a significant revenue source for the facility types we analyzed. Finally, our analysis is a “best case” scenario if every non-OTP outpatient SUD treatment program integrated methadone treatment and therefore our estimates reflect an upper bound of spatial accessibility. Further geospatial analysis could identify key SUD treatment programs to be prioritized for integration efforts and further implementation research could identify SUD treatment program characteristics that are associated with successful uptake of integration.

In conclusion, we found substantial spatial accessibility of OTPs by public transit in NYC. Expanding methadone treatment to non-OTP outpatient SUD treatment programs has the potential to improve the spatial accessibility of methadone treatment and improve treatment outcomes.
